# Hydralazine-Induced Anti-neutrophil Cytoplasmic Antibody (ANCA)-Associated Vasculitis

**DOI:** 10.7759/cureus.47656

**Published:** 2023-10-25

**Authors:** Victoria Echevarria, Ednord Pierre, Jorge Quiros, Parham Eftekhari

**Affiliations:** 1 Internal Medicine, American University of the Caribbean School of Medicine, Sint Maarten, SXM; 2 Internal Medicine, Broward Health Medical Center, Fort Lauderdale, USA; 3 Nephrology, Broward Health Medical Center, Fort Lauderdale, USA

**Keywords:** hydralazine, drug-induced vasculitis, anti-histone antibody, hydralazine associated vasculitis, anca associated vasculitis

## Abstract

Anti-neutrophil cytoplasmic antibody (ANCA)-associated vasculitis comprises several conditions involving vascular destruction that extends into tissue necrosis. There are several autoimmune and environmental causes implicated in the disease progression; among these is drug-induced vasculitis caused by hydralazine use. Hydralazine-induced vasculitis is an uncommon potential complication of the medication and can progress to multisystem involvement and eventually advance to end-organ damage and renal failure. Our patient presented with symptoms of lower extremity edema, dyspnea, and a nonproductive cough eventually resulting in the identification of hydralazine-induced ANCA-associated vasculitis with hypocomplementemia and positive anti-histone antibody. Due to the prevalence of hydralazine as a cardiac drug, physicians managing patients on the medication should have a high index of suspicion of the potential for vasculitis in order to promote prompt diagnosis and treatment of the ANCA-vasculitis.

## Introduction

Anti-neutrophil cytoplasmic antibody (ANCA)-associated vasculitis describes several disorders affecting small and medium-sized blood vessels which result in widespread vascular damage and tissue necrosis [1,2]. The pathogenesis of the conditions involves the formation of autoantibodies to neutrophil-containing proteins such as leukocyte proteinase 3 (PR3-ANCA) or myeloperoxidase (MPO-ANCA) which form major components within granules of monocytes. Although the pathogenesis is not completely understood, the progression of vascular involvement spans several different body systems and has a diverse symptom presentation [1,2]. ANCA vasculitis is known to have ear, nose, throat, lung, and renal involvement, to name a few. The conditions are associated with not only autoimmune causes but have also been linked to infections, environmental factors such as silica, as well as drugs like propylthiouracil, hydralazine, and Isoniazid [1]. Hydralazine-induced vasculitis, in particular, can develop in patients taking the medication for the treatment of chronic hypertension, hypertensive emergency, and heart failure. Hydralazine is a medication used commonly in cardiac patients for blood pressure management; it is a direct vasodilator that induces arterial dilation via the inhibition of calcium release from the sarcoplasmic reticulum and phosphorylation within arterial smooth muscle cells [3]. In this article, we present a case of hydralazine-induced ANCA vasculitis to demonstrate the potentially fatal and often overlooked side effects of this medication.

## Case presentation

The patient is a 71-year-old female with a past medical history of atrial fibrillation. The status post Watchman device placement was pernicious anemia, hypertension, hypothyroidism, and hypercholesterolemia, with otherwise no history of chronic kidney disease. She presented to our emergency department (ED) with complaints of bilateral lower extremity edema, progressively worsening dyspnea at rest, and non-productive cough with onset two and a half weeks prior to admission. Four days prior to arrival at the ED, the patient was admitted to a hospital in the United Kingdom with similar complaints. The diagnostic workup was negative for pulmonary embolism. The patient was advised to follow up with a nephrologist due to elevated blood area nitrogen (BUN) and creatinine levels. Home medications included hydralazine 50 mg twice daily. Hydralazine was started approximately 20 months prior to admission as an adjuvant for hypertension. 

On initial presentation, vital signs showed blood pressure of 150/90 mmHg and a heart rate of 101 bpm. The patient was afebrile and saturating 98% on ambient air. The initial laboratory workup is shown below in Table 1. Further workup included a chest x-ray, which showed no active pulmonary process, and a Doppler ultrasound of the bilateral lower extremities which was negative for deep vein thrombosis. Echocardiogram showed an ejection fraction of >55%, and biatrial dilatation. Lung ventilation/perfusion scanning resulted in a low probability of acute pulmonary embolism. 

**Table 1:** Laboratory workup CBC, complete blood count; CMP, comprehensive metabolic panel; FEU, fibrinogen equivalent units; Ab, antibody; hpf, high power field

Lab test	Patient value	Reference range
CBC		
Hemoglobin	8.4 g/dL	12.1 to 15.1 g/dL
Hematocrit	26.3%	36% to 48%
Mean corpuscular volume	94.3 fL	80 - 95 fL
Mean corpuscular hemoglobin concentration	31 g/dL	33 - 36 g/dL
White blood cell count	4.5 x10^3/uL	4.5 - 11.0 × 10^9^/L
Absolute neutrophil count	3.61x10^3/uL	1.4 - 7 x 10E3/uL
Absolute lymphocytes	0.49x10^3/uL	1.0 – 4.5 x 10^3/uL
CMP		
Blood urea nitrogen	25 mg/dL	7 - 20 mg/dL
Creatinine	2.3 mg/dL	0.7 - 1.3 mg/dL
Potassium	3.1 mmol/L	2.7 - 4.5 mg/dl
Specific testing		
Brain natriuretic peptide	669 pg/mL	<100 pg/mL
D-dimer	4.0 ug/mL FEU	<0.50 mg/L
Troponin I x2	0.05-0.06 ng/mL; negative	0 - 0.04 ng/mL
Serology		
Antinuclear Abs	Negative	Negative
Scleroderma Ab (SCL 70) level	<0.1	≤0.9 AI
Anti-myeloperoxidase (MPO) Ab level	>800	<1.0 AI
Anti proteinase 3 (PR 3) Ab level	<0.1	≤20.0 U
dsDNA Ab level	6 IU/mL	≤4 IU/ mL
Histone Ab level	2.9 U	0.0–0.9 U
C3 level	60 mg/dL	88 - 201 mg/dL
C4 level	7 mg/dL	15 - 45 mg/dL
Urinalysis		
Red blood cells	308/hpf	<4 RBC/hpf
White blood cells	15/hpf	<2-5 WBC/hpf
Urine protein	30 mg/dl	0 - 14 mg/dL
Creatinine	28.9 mg/dL	0.7 - 1.2 mg/dL
Sodium	58 mmol/L	< 40 mmol/L

**Figure 1:**
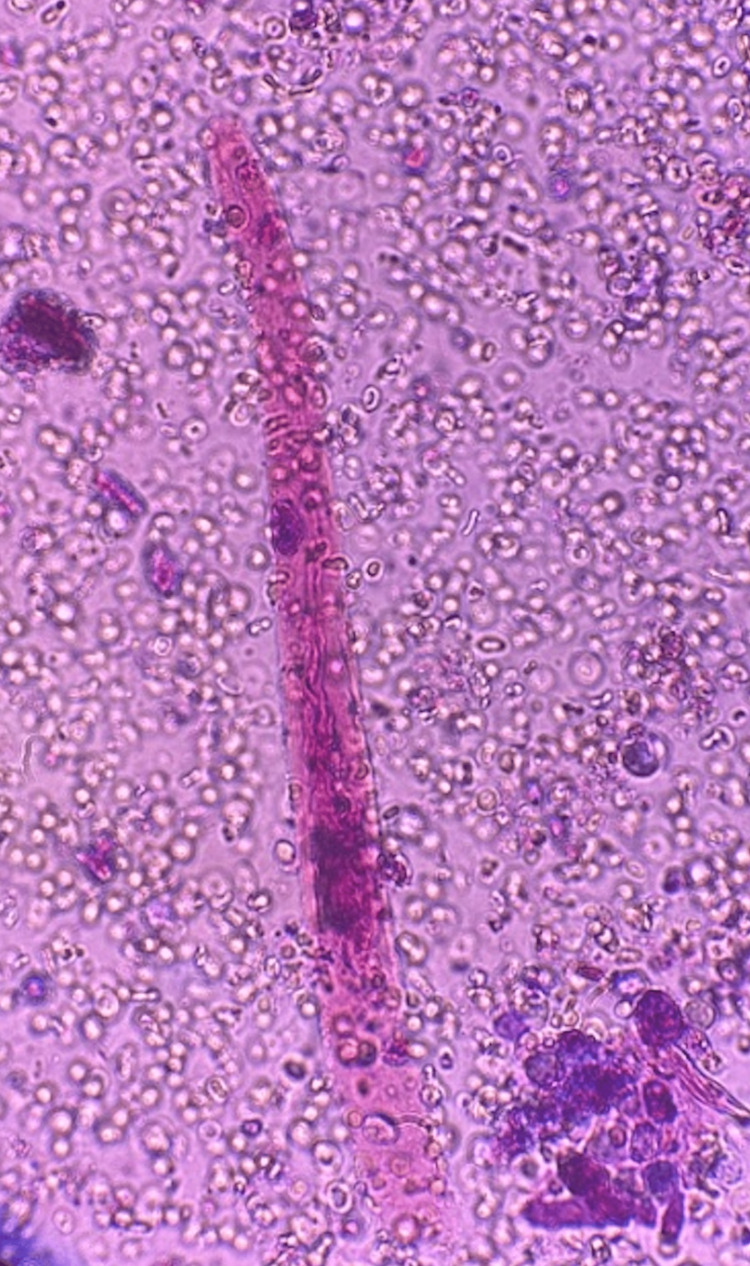
Urinalysis with brightfield illumination revealing red blood cell casts with dysmorphic red blood cells indicating glomerulonephritic process.

Urinalysis was significant for hematuria with an RBC of 308/hpf, WBC of 15/hpf, and urine protein of 30 mg/dl. Urine creatinine was 28.9 mg/dL, urine sodium 58 mmol/L, and urine total protein 43 mg/dl. Our differential diagnosis was inclusive of cryoglobulinemia, systemic lupus erythematosus (SLE) nephritis, and post-infectious glomerulonephritis. Urine microscopy spun by ultrasound revealed RBC casts suggestive of nephritic syndrome (Figure 1). After urinary RBC casts were seen, complement levels were assessed for possible vasculitis. Complement levels (C3 and C4) were 60 mg/dl and 7 mg/dl, respectively, depicting hypocomplementemia. Further investigations revealed a positive histone antibody and MPO-ANCA (serology in Table 1). Ten weeks prior, outpatient laboratory workup reviewed showed a baseline BUN of 15 mg/d and creatinine of 0.8 mg/dL. 

**Figure 2:**
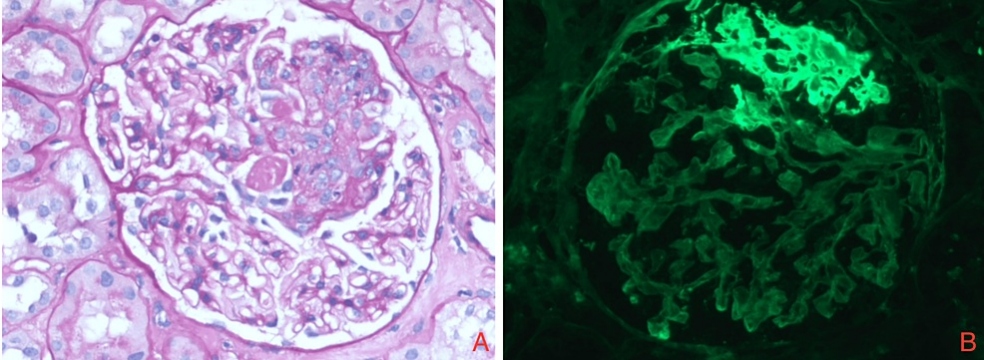
Renal biopsies (A) with hematoxylin and eosin stain visualized under light microscopy showing inflammatory cells and fibrocellular deposition, and (B) with glomerular and capillary wall stain visualized under immunofluorescence microscopy showing immune complex deposition.

Patient clinical findings of hypertension, proteinuria, hematuria, hypocomplementemia, and renal failure suggest nephritic syndrome. ​​​This prompted us to obtain renal biopsies which showed inflammatory cells and fibrocellular deposition in hematoxylin and eosin stain (under light microscopy) as well as immune complex deposition in glomerular and capillary wall staining (under immunofluorescence microscopy) (Figures 2A-2B).** **The diagnosis of rapidly progressive glomerulonephritis was made. Renal ultrasound findings showed diffusely increased renal echogenicity bilaterally. Testing for hepatitis B surface antigen, hepatitis B core IgM, hepatitis C antibodies, and hepatitis A IgM were negative. HIV antibody antigen was negative. Rapid plasma reagin (RPR) titer was 1:1, *Treponema* syphilis was non-reactive.

## Discussion

Drug-induced ANCA-associated vasculitis (AAV) is characterized by necrotizing vascular inflammation which affects small and medium vessels [2]. Although the exact pathology for hydralazine-induced AAV is unknown, there have been several theories for its proposed mechanism including one in which neutrophil apoptosis occurs as a result of cytotoxic product release once hydralazine accumulates in neutrophils and binds to myeloperoxidase [3]. AAV serologic evaluation typically reveals anti-nuclear cytoplasmic antibodies (ANCA), hypocomplementemia, and anti-histone antibodies in addition to high-titer MPO-ANCA [4]. Those with positive serology present with symptoms of myalgias, fever, and serositis [5]. 

Hydralazine is widely prescribed in the United States. In 2020, 6.6 million prescriptions were estimated to be prescribed to 1.6 million patients. Complications include drug-induced autoimmunity such as hydralazine-induced lupus erythematosus, cutaneous and renal vasculitis, as well as glomerulonephritis [6]. 

The diagnosis of hydralazine-induced ANCA-associated vasculitis was established in our patient with the presence of the following: positive anti-histone antibodies, elevated anti-myeloperoxidase antibodies, hypocomplementemia (C3 and C4), urinary casts, hematuria, proteinuria, and kidney biopsy. This case is distinguished from other documented cases in the literature due to the low complement levels of both C3 and C4. In a study performed by Santoriello et al. in 2021, 66 participants with confirmed hydralazine-induced ANCA-associated glomerulonephritis, found that 58% (38 out of 66 participants) exhibited hypocomplementemia, out of which only 28 participants had decreased in both C3 and C4 levels [4]. 

Hydralazine-induced lupus has been thought to be dose-dependent with an incidence of 5.4% in patients taking 100 mg daily, and 10.4% in those taking 200 mg daily for 3 years who were found to be slow acetylators [3]. Hydralazine-induced AAV appears to be a rare complication, most commonly involving the kidneys and less frequently the respiratory system causing a more severe presentation. In a study by Choi et al., 30 subjects were selected with anti-MPO antibodies titers which were more than twelve times the median titer out of 250 subjects in total [7]. It was found that 33% of those subjects studied had been exposed to hydralazine and had predominantly renal involvement. Idiopathic ANCA vasculitis and hydralazine-induced vasculitis have overlapping presentations and may be difficult to clinically differentiate, but the latter is typically resolved by discontinuing the suspected drug agent.

Some risk factors associated with increased risk for hydralazine-induced ANCA-associated vasculitis are a cumulative dose of more than 100 mg of hydralazine per day, female gender, and thyroid disease [3,8]. No effective guidelines have been established for the management of hydralazine-induced vasculitis due to the rarity of the disease. Primary intervention has involved the discontinuation of the culprit drug along with the administration of immunosuppressive therapy based on disease severity [9-11]. The patient discussed in this report was treated with two infusions of Rituximab 1000 mg and high-dose steroids. Follow-up visits showed resolution of symptoms and return to baseline renal function on follow-up three months later (BUN 20 mg/d, creatinine 0.9 mg/dL). After the high-dose steroid was tapered off, repeat labs revealed an anti-MPO of 5.9 AI and an anti-histone antibody <1. The clinical education of both clinicians and patients regarding the potential side effects of Hydralazine use is critical. Further studies into the association, management, and prevention of hydralazine and its potential role in ANCA-induced vasculitis are warranted.

## Conclusions

Hydralazine is a commonly prescribed medication for blood pressure control in cardiac patients. Although rare, ANCA-associated vasculitis may be caused by hydralazine use, especially in patients taking high doses of the medication. In this paper, we aim to encourage physicians to consider hydralazine as a cause of ANCA-associated vasculitis in patients presenting with glomerulonephritis who have a history of hypertension. In the patient presented, the diagnosis was made based on the display of symptoms as well as positive histone antibody and MPO-ANCA, casts on urinalysis, proteinuria, and hematuria. Physicians should remain suspicious of hydralazine as a cause of ANCA-associated vasculitis, as the early diagnosis and discontinuation of hydralazine are crucial for a favorable prognosis.
